# Adult Lead Exposure: Time for Change

**DOI:** 10.1289/ehp.9782

**Published:** 2006-12-22

**Authors:** Brian S. Schwartz, Howard Hu

**Affiliations:** 1 Departments of Environmental Health Sciences and Epidemiology, Johns Hopkins Bloomberg School of Public Health, Baltimore, Maryland, USA; 2 Department of Medicine, Johns Hopkins School of Medicine, Baltimore, Maryland, USA; 3 Department of Environmental Health Sciences, University of Michigan School of Public Health, Ann Arbor, Michigan, USA

**Keywords:** adults, biomarker, cumulative dose, epidemiology, lead, lead poisoning, OSHA regulations, tibia bone lead, U.S. Occupational Safety and Health Administration, worker protection, X-ray fluorescence, XRF

## Abstract

We have assembled this mini-monograph on adult lead exposure to provide guidance to clinicians and public health professionals, to summarize recent thinking on lead biomarkers and their relevance to epidemiologic research, and to review two key lead-related outcomes, namely, cardiovascular and cognitive. The lead standards of the U.S. Occupational Safety and Health Administration are woefully out of date given the growing evidence of the health effects of lead at levels of exposure previously thought to be safe, particularly newly recognized persistent or progressive effects of cumulative dose. The growing body of scientific evidence suggests that occupational standards should limit recent dose to prevent the acute effects of lead and separately limit cumulative dose to prevent the chronic effects of lead. We hope this mini-monograph will motivate renewed discussion of ways to protect lead-exposed adults in the United States and around the world.

Public health and regulatory efforts in the United States have achieved great successes over the last 40 years regarding environmental lead exposure. Chronological trend data from the National Health and Nutrition Examination Surveys have documented a decline in average adult blood lead levels from approximately 15 μg/dL in the 1970s to the current 1–2 μg/dL (Annest et al. 1983; Muntner et al. 2005; Pirkle et al. 1998). Similar changes have occurred in children in the context of recommendations by the U.S. Centers for Disease Control and Prevention CDC) for a progressive decline in the level of concern of lead in a child’s blood that should trigger other specific actions (from 40 to 30 μg/dL in 1975, 30 to 25 μg/dL in 1985, and 25 to 10 μg/dL in 1991). This trend was driven by a steady accumulation of research that demonstrates adverse effects in children at progressively lower levels of exposure and reflects the success of a number of regulatory actions that removed lead from gasoline and many other consumer products such as paints and solder used for plumbing and food cans.

In contrast, despite a growing body of research that demonstrates adverse effects in adults at progressively lower levels of exposure, the lead standards of the U.S. Occupational Safety and Health Administration (OSHA), promulgated in final form for general industry (29 CFR 1910.1025; OSHA 2006a) in 1978 and for construction (29 CFR 1926.62; OSHA 2006b) in 1993 have not changed in their allowance that workers can attain blood lead levels up to 40 μg/dL for their working lifetimes. OSHA, relying on studies at that time, developed these standards when most workers started employment in lead-exposed jobs with average blood lead levels in the 10- to 15-μg/dL range because of high environmental exposures. As such, the research that formed the basis of the health protection goals in the standards was primarily from the 1960s and 1970s and earlier. This lack of change has no doubt contributed to a trend in occupational lead exposure that has not declined as steeply as environmental lead exposure. In 2002, a total of 10,658 adults with blood lead levels of ≥ 25 μg/dL were reported to the CDC by 35 states (CDC 2004). Of these, 1,854 had levels of ≥ 40 μg/dL. These numbers are likely to underestimate the true magnitude of the problem because many workers who should be in employer-sponsored surveillance programs to follow their blood lead concentrations are not well monitored (Nelson and Kaufman 1998; Papanek et al. 1992; Rudolph et al. 1990).

The recognition of the inadequacy of the OSHA lead standards is not new (Landrigan et al. 1990; Silbergeld et al. 1991). However, the quality of the evidence demonstrating that lead exposure at levels below the OSHA standard is associated with significant adverse effects has dramatically improved. Epidemiologic methods in general and as applied to research on the health effects of lead have evolved considerably during this time, with more rigorous attention to study design, subject selection, causal pathways, lead dose, lead biomarkers, and genetic susceptibility factors, particularly regarding such issues as timing and accumulation of dose and the development of acute and chronic health effects. Acute health effects due to recent dose are thought more likely to be reversible in nature, whereas chronic health effects due to cumulative dose are thought more likely to be irreversible in nature. It is thus critical to address these issues and consider them in research studies.

## Purposes of the Mini-Monograph

This mini-monograph is meant to serve several purposes. First, it provides recommendations to the medical and public health communities in the medical management of adults with lead exposure. Second, it is a discussion of the state-of-the-art thinking on epidemiologic studies of the health effects of lead in adults, particularly regarding the use and interpretation of lead biomarkers, study population and design issues, and complex causal pathways involving how lead affects human health. Third, the mini-monograph includes two systematic reviews (Navas-Acien et al. 2007; Shih et al. 2007) of epidemiologic evidence regarding two of the most important health outcomes associated with lead, specifically, the effects on the cardiovascular and central nervous systems.

Currently, no other similar compilations have summarized recent scientific findings and their relevance to clinical and public health practice regarding lead-exposed adults. Furthermore, although many authors have increasingly recognized the importance of recent and cumulative lead dose as predictors of adverse health effects, especially for lead, no documents have summarized these issues and their relevance to protection of lead-exposed adults, or made recommendations for protection that use limits on both recent dose and cumulative dose. Finally, it is a call to action. The OSHA lead standards provide inadequate protection to lead workers; the existing evidence is compelling that we are subjecting lead workers to too large a burden of risk for both acute and chronic health effects.

We have assembled five articles, including the present article, into this mini-monograph on adult lead exposure (Hu et al. 2007; Kosnett et al. 2007; Navas-Acien et al. 2007; Shih et al. 2007). As described below, the mini-monograph was motivated largely by the work of a national expert panel on adult lead exposure that spent over 2 years discussing these issues. In this first article, we describe the history and limitations of the OSHA lead standard, the process of the expert national panel, the purpose of the other articles in the mini-monograph, and provide some personal recommendations on what is required to protect workers from the health effects of lead.

## Process for Development of the Guidelines

The first impetus for the articles assembled in the mini-monograph was from the Adult Blood Lead Epidemiology and Surveillance (ABLES) Program, a National Institute for Occupational Safety and Health (NIOSH) endeavor currently operating in 38 states. In 2000, ABLES formed an ad hoc Committee for the Development of Adult Blood Lead Level Medical Management Guidelines and a draft document was produced (unpublished). The Association for Occupational and Environmental Clinics (AOEC) agreed to sponsor the next steps in the review and revision of the draft document, and obtained federal funding in support of this activity. AOEC next assembled a panel of 13 experts with training and experience in the areas of lead toxicology, epidemiology, occupational medicine, occupational health nursing, industrial hygiene, and public health policy and practice, from academic institutions, government, labor organizations, and industry to review the document. Panel members included (in alphabetical order) Rose Goldman, Dana Headapohl, Karen Hipkins, Howard Hu, Michael Kosnett, Barbara Materna, Pamela Reich, Stephen Rothenberg, Brian Schwartz, Eugene Shippen, Richard Wedeen (Panel Chair), Laura Welch, and Alan Woolf. Kathy Kirkland (Executive Director of the AOEC), coordinated the activities of the panel.

The panel met in March 2003 in Washington, DC, and then held a series of conference calls through 2005 to make revisions to the document. At the onset, the panel chose to focus on health-based recommendations and not to explicitly consider feasibility of implementing these guidelines. The panel also generally decided not to explicitly consider socioeconomic considerations for lead workers, for example, if a cumulative lead dose limit required workers currently in the lead-using industries to discontinue all further work in lead and thus had to leave their jobs. Measures designed to protect health may incur unacceptable costs to individual workers and industry, but the considerations of panel members and authors of the articles in the mini-monograph were solely motivated by scientific evidence regarding health. Conclusions stated in all articles in the mini-monograph are not intended to be enforceable standards, which by law must reflect feasibility and experience gained under OSHA and other health and safety laws. The third article (Kosnett et al. 2007) in the mini-monograph was prepared by a subgroup of panel members (8 of 13 members) who were interested in and willing to continue to work on these issues after the work of the panel ended. The article is not a systematic review but rather panel members relied on selected, influential published articles within the extant literature as well as experience and expert opinion to come to consensus in reaching the article’s recommendations. However, it represents only the opinions of its authors.

Overall, there was a remarkable degree of consensus among panel members. The two industry representatives on the panel voiced a number of concerns during the process, but other panel members generally agreed on all but a few points. Although the panel made considerable progress in reaching consensus, a number of challenges to its process must be acknowledged. Funding was available only for one face-to-face meeting. This meeting highlighted many difficult issues that needed continued analysis and discussion, but all further discussion was only available by conference call. In contrast to typical Institute of Medicine (IOM) committees charged with reviewing scientific evidence and making recommendations for public health or clinical medicine (National Academy of Sciences 2006), our committee had more limited resources and lacked an agreed upon, standardized approach to review of the evidence and presentation of its analysis and summary, as is used by IOM committees. Despite these limitations, the majority of panel members supported the final document in the mini-monograph, which provides clear recommendations on a number of management approaches for lead-exposed adults that we believe will be useful to public health and clinical practitioners.

## Brief Review of Requirements of the OSHA Lead Standards

A review of the OSHA lead standards and their preambles allows several conclusions. First, the standards emphasize prevention of acute symptoms in several organ systems and prioritize other measurable health effects in a way that is likely to be considered differently today. Most health effects that are considered are of relatively short latency and are more likely to occur after high-level, short-term exposures. For example, there is extensive discussion of the hematopoietic system in which measurable health effects occur with short latency after moderate to high lead exposures and very little consideration of long-latency, chronic health effects. Such chronic health outcomes as cognitive dysfunction, hypertension risk, and renal dysfunction after long-term, low-level exposures were not considered in any substantive detail. So, while the OSHA standards mainly focused on prevention of symptoms, hematopoietic outcomes, and renal dysfunction associated with high-level exposures, the highest priority concerns of today would be cognitive decline, hypertension and other cardiovascular outcomes, long-latency renal disease, and reproductive outcomes.

Second, the standards considered the level of lead in whole blood to be the key lead bio-marker and, although not explicitly discussed, blood lead was generally used only as a measure of relatively recent dose. There was no consideration of cumulative dose or long-term lower-level exposures despite the fact that many health outcomes associated with environmental exposures are due to cumulative dose (e.g., environmental tobacco smoke and lung cancer) and the fact that lead was known to accumulate in bone and thus has long residence times in the body. Health physicists were developing and validating X-ray fluorescence (XRF) systems for measurement of lead in bone at that time, but certainly by the late 1980s or early 1990s, there was extensive experience with measurement of lead in bone by cadmium-109–induced K-shell XRF, if not widespread availability. Third, there was no consideration about whether health effects could progress after cessation of occupational exposure. Finally, there was little consideration of susceptible subgroups such as older individuals or those with certain genetic polymorphisms. In fairness, little was known about genetic susceptibility to lead poisoning in the 1970s, but a large number of studies have been published on the topic since that time. Any new recommendations for occupational lead exposure standards should recognize that there are susceptible subgroups and limits should protect the most susceptible workers.

Under the OSHA standards, medical surveillance is an essential part of an employer’s lead safety program and includes biological monitoring with periodic blood lead testing, medical evaluation, and treatment if needed, and intervention to prevent or control exposure levels. Employers must offer medical surveillance to workers if airborne lead exposure is 30 μg/m^3^ (8-hr time-weighted average) or higher for > 30 days/year. Although slight differences exist in the two OSHA lead standards, OSHA requires that workers in general industry be removed from further lead exposure if a single blood lead level is ≥ 60 μg/dL, or if three determinations over 6 months average ≥ 50 μg/dL, until levels decline to < 40 μg/dL. An important implication of these limits is that, because OSHA accepts a blood lead level of 40 μg/dL for a working lifetime (40 years), a cumulative blood lead index of 1,600 μg-years/dL (calculated as the average blood lead level multiplied by the years over which it is averaged, or, more accurately as the area under the curve of blood lead versus time) is an acceptable cumulative dose. As discussed in greater detail in the second article (Hu et al. 2007) in the mini-monograph, this cumulative blood lead index would result in a bone lead level (in the tibia) at the end of employment of 80–160 μg lead/g bone mineral. This mini-monograph provides extensive evidence that this cumulative dose is associated with substantial adverse health risks.

If new OSHA standards are developed and promulgated, there will arise a number of complex and challenging issues. For example, because of the significant reduction of lead in the general environment, new workers will enter lead jobs with very low blood lead levels, whereas others who have worked with lead or who are older will have much higher blood lead levels and body burdens. This may motivate employers to hire younger workers and terminate older workers. This mini-monograph does not address these complexities and leaves consideration of these issues to future efforts.

## New Evidence on the Health Effects of Lead

The growth in knowledge about the health effects of lead has been dramatic since the promulgation of the OSHA lead standards. For example, in 1995, Balbus-Kornfeld et al. (1995) reported there was no evidence for or against the hypothesis that cumulative lead dose caused cognitive dysfunction or decline, because no epidemiologic studies had estimated cumulative dose and the four existing prospective studies were small, with relatively low follow-up rates, and of relatively short duration. As discussed in the second article (Hu et al. 2007) in the mini-monograph, longitudinal studies provide critical evidence because confounding is less likely to invalidate inferences and such studies allow differentiation of reversible, persistent, and progressive health effects, which also requires that occupational exposures have ended. Since that time, at least 20 papers have been published (or are in press) that document studies measuring cognitive function, blood lead, and bone lead, thereby allowing more careful consideration of recent and cumulative dose than in earlier studies. This dramatic growth in scientific evidence has not been confined to cognitive function; similar, if not greater, growth has occurred regarding other important health effects of lead exposure such as hypertension and other cardiovascular outcomes (Lustberg and Silbergeld 2002) and renal disease (Weaver et al. 2005). These new studies allow much better evaluation of such key issues as recent, intermediate-term, and cumulative dose; acute and chronic health effects; and reversibility, persistence, or progression of health effects.

## Recommendations for a New OSHA Standard

In our opinion, which does not necessarily represent the view of the expert panel, we believe that lead poisoning must be thought about as a chronic disease. Once a significant lead body burden has accumulated, the health effects are likely to be progressive and, to a large degree, irreversible. Like diabetes and other chronic diseases, these health conditions can be managed, but some health consequences may not be preventable after a cumulative dose threshold is exceeded. We must prevent cumulative dose, not just follow blood lead levels. We must focus on prevention of long-term, progressive health effects, such as cognitive decline, and the increased risk of cardiovascular and circulatory mortality. We must also prevent the shorter latency health effects of recent dose, as reflected in blood lead levels, such as elevations in blood pressure and the cognitive dysfunction present at only moderate blood lead levels. We must acknowledge that there are likely to be susceptible subgroups, as suggested by recent studies demonstrating worse lead-associated outcomes in persons with certain polymorphisms in the apolipoprotein E (Stewart et al. 2001), vitamin D receptor (Lee et al. 2001a, 2001b; Schwartz et al. 2000a, 2000b), Na^+^,K^+^-ATPase (Glenn et al. 2001), and δ-aminolevulinic acid genes (Bergdahl et al. 1997; Schwartz et al. 2000a, 1997; Wetmur 1994; Wetmur et al. 1991; Wu et al. 2003), protein kinase C phenotypes (Hwang et al. 2002), and persons with other common chronic diseases such as type II diabetes (Tsaih et al. 2004), and promulgate lead standards that prevent adverse health outcomes in these most susceptible subgroups.

We recommend that OSHA modify the lead standards to prevent both the acute health effects of recent dose and the chronic health effects of cumulative dose. It should be noted that the members of the panel considered the use of tibia lead levels and cumulative blood lead index in detail, but concluded they could not recommend routine clinical use of the former because of limited availability and the on-going but incomplete development of standardized protocols for intercalibration—the latter because of concerns about logistics and practicality. However, the panel recognized that the main advances in epidemiologic research on the health effects of lead in the past two decades have been recognition of the importance of cumulative dose and that a single or few blood lead levels do not estimate cumulative dose. Accumulation of dose in this context is a critically important concept that has not been generally considered in occupational safety and health regulations to date and is the motivation for many of the recommendations in this mini-monograph.

We would favor limits that keep blood lead levels < 20 μg/dL to prevent the acute effects of recent dose. For the prevention of the chronic health effects of cumulative dose, the available evidence suggests that tibia lead levels should not be allowed to exceed 15 μg lead/g bone mineral; this could also be achieved by maintaining the cumulative blood lead index below approximately 200–400 μg-years/dL (equivalent to an average blood lead level of 20 μg/dL for 10–20 years or of 10 μg/dL for 20–40 years). Unfortunately, other scientists and public health professionals made similar recommendations more than 15 years ago (Landrigan et al. 1990; Silbergeld et al. 1991), and little has resulted. We hope this mini-monograph will have a larger impact on policy.

These recommendations require more stringent exposure limits than currently exist in the OSHA standards and a tighter link between exposure and dose in surveillance programs. The OSHA permissible exposure limit of 50 μg/m^3^ was based on industry claims of feasibility for engineering and ventilation controls, but current technologies are better than those that were available in the 1970s. However, the lower limits may require greater reliance on respirators, which will have implications for both workers and employers.

## Organization of the Mini-Monograph

The mini-monograph consists of four other articles:

The article by Hu et al. (2007) is a review of issues surrounding epidemiologic methods of particular relevance to studies of the health effects of lead, lead exposure and dose, and lead biomarkers. These issues are relevant to each of the other articles in the mini-monograph.

Kosnett et al. (2007) summarize recommendations for management of adults with lead exposure. This article was prepared by a majority of members of an expert panel (8 of 13 members) and offers advice to public health professionals and clinicians in the medical and nonmedical management of lead-exposed adults. The expert panel’s final document also went to the AOEC for additional editing, which resulted in the greatly shortened manuscript on the AOEC website AOEC 2006). The manuscript and recommendations on the AOEC website were not written by panel members; rather, they were rewritten by a small committee of AOEC members.

Navas-Acien et al. (2007) review the epidemiologic evidence evaluating lead exposure and cardiovascular outcomes. Cardiovascular outcomes were critical to the deliberations of the panel, but such detailed analysis of studies could not be incorporated into the panel’s main article. Here, the authors consider this evidence in detail. They summarize the review articles on blood pressure and hypertension, but go into great detail on cardiovascular outcomes other than blood pressure and hypertension, including important clinical ones (e.g., myocardial infarction).

The review by Shih et al. (2007) is a discussion of the epidemiologic evidence evaluating lead exposure and cognitive outcomes. Like cardiovascular outcomes, many panel members considered cognitive outcomes to be from a critical target organ and prevention of these outcomes was an important motivation. Here, the authors considered only articles that measured blood lead, bone lead, and cognitive outcomes, so the key issues discussed by Hu et al. (2007) could be evaluated.

Other outcomes were also considered by the panel but additional manuscripts were not solicited for the mini-monograph for several reasons. First, at the time of preparation of the mini-monograph, the U.S. Environmental Protection Agency (U.S. EPA) was in the process of updating its Air Criteria Document for Lead (U.S. EPA 2005) and systematic reviews of evidence by organ system will be a result. We are aware of several authors of chapters for that document who have already made plans to submit their chapters for publication in other specialty journals (Ekong et al. 2006). Finally, the CDC has convened a national panel on lead and reproductive outcomes (CDC 2006), so we did not solicit a reproductive chapter in advance of that effort.
